# Estimation of Glomerular Filtration Rate in Obese Patients: Utility of a New Equation

**DOI:** 10.3390/nu15051233

**Published:** 2023-02-28

**Authors:** Pehuén Fernández, María Laura Nores, Walter Douthat, Javier de Arteaga, Pablo Luján, Mario Campazzo, Jorge de La Fuente, Carlos Chiurchiu

**Affiliations:** 1 Nephrology Service, Hospital Privado Universitario de Córdoba, Córdoba 5000, Argentina; 2 Graduate Career in Nephrology, Instituto Universitario de Ciencias Biomédicas de Córdoba (IUCBC), Córdoba 5000, Argentina; 3 Facultad de Matemática, Astronomía, Física y Computación, Universidad Nacional de Córdoba, Córdoba 5000, Argentina; 4 Clinical Biochemistry Laboratory, Hospital Privado Universitario de Córdoba, Córdoba 5000, Argentina; 5 Bariatric Surgery Program, Hospital Privado Universitario de Córdoba, Córdoba 5000, Argentina

**Keywords:** glomerular filtration rate, obesity, creatinine-based equations, kidney function tests, iothalamate meglumine, chronic kidney disease

## Abstract

There is no consensus on the best equation to estimate glomerular filtration rate (eGFR) in obese patients (OP). Objective: to evaluate the performance of the current equations and the new Argentinian Equation (“AE”) to estimate GFR in OP. Two validation samples were used: internal (IVS, using 10-fold cross-validation) and temporary (TVS). OP whose GFR was measured (mGFR) with clearance of iothalamate between 2007/2017 (IVS, *n* = 189) and 2018/2019 (TVS, *n* = 26) were included. To evaluate the performance of the equations we used: bias (difference between eGFR and mGFR), P30 (percentage of estimates within ±30% of mGFR), Pearson’s correlation (r) and percentage of correct classification (%CC) according to the stages of CKD. The median age was 50 years. Sixty percent had grade I obesity (G1-Ob), 25.1% G2-Ob and 14.9% G3-Ob, with a wide range in mGFR (5.6–173.1 mL/min/1.73 m^2^). In the IVS, AE obtained a higher P30 (85.2%), r (0.86) and %CC (74.4%), with lower bias (−0.4 mL/min/1.73 m^2^). In the TVS, AE obtained a higher P30 (88.5%), r (0.89) and %CC (84.6%). The performance of all equations was reduced in G3-Ob, but AE was the only one that obtained a P30 > 80% in all degrees. AE obtained better overall performance to estimate GFR in OP and could be useful in this population. Conclusions from this study may not be generalizable to all populations of obese patients since they were derived from a study in a single center with a very specific ethnic mixed population.

## 1. Introduction

Obesity is currently an important public health problem, its prevalence is increasing year after year throughout the world, and Latin America does not seem to be the exception [1,2]. The relationship between obesity and type 2 diabetes, hypertension, dyslipidemia, coronary heart disease, cerebrovascular accident and some types of cancer is well known [3,4]. Some authors suggest that obesity also increases the risk of initiation and progression of chronic kidney disease (CKD) [5,6,7], not only due to its relationship with its already known traditional risk factors, but also due to a direct effect on renal structure [5,8,9,10].

It is important to know as precisely as possible the glomerular filtration rate (GFR) in patients with obesity, since they belong to a risk group. The most precise method to measure GFR is by urinary inulin clearance [11], although measurement of urinary clearance with iothalamate yields similar results and is also considered the gold standard [12]. These methods are complex, impractical, expensive, and poorly available. In clinical practice, endogenous markers such as serum creatinine (SCr) are commonly used to estimate GFR using different formulas. Estimating GFR from SCr in the obese is challenging, since there may be determinants that affect SCr generation, such as diet, nutritional status, extreme body size, and hidden relative sarcopenia [13,14]. For this reason, SCr levels can be erratic in estimating GFR. The formulas commonly used in clinical practice are not calibrated for use in this population. In addition, these have been developed in populations with ethnic groups other than the typical Latin American race (mixture between natives, Spanish and Italians). For this reason, we developed a new equation using novel statistical strategies, called AE (“Argentinian Equation”), in which only subjects of Latin American origin were included in their entirety, and other predictor variables were added that could improve the prediction. There is currently no consensus on which is the best equation to estimate GFR in subjects with obesity. The aim of this study was to evaluate the performance of the currently available formulas and the new AE for estimating GFR in obese patients.

## 2. Materials and Methods

A cross-sectional study was carried out. Two samples were used to validate the equations: the internal validation sample (IVS) and the temporary validation sample (TVS).

For IVS, all individuals of Latin American origin were consecutively included, whose GFR was measured using iothalamate urinary clearance, at the Hospital Privado Universitario de Córdoba, between January 2007 and December 2017. Indications for assessment were: suspected or established renal dysfunction, renal risk or before kidney donation. The exclusion criteria were: minors, ethnic groups other than Latin American, history of cirrhosis, decompensated heart failure, more than one GFR measurement (only the first measurement was used), incorrect urine collection, hospitalized, those who presented determinations of serum creatinine, urea and/or albumin, separated more than 7 days before or after the GFR measurement. Absence of data in the single-renal variable, and all subjects with a body mass index (BMI) < 30 kg/m^2^. For the TVS, the same inclusion and exclusion criteria were used, only that it was created after the IVS and subjects were included between January 2018 and July 2019.

Gender, age (years), BMI (Kg/m^2^), history of hypertension, diabetes mellitus, and single kidney were recorded. The values of SCr (mg/dL), urea (mg/dL), albumin (gr/L) and GFR measured with urinary clearance of iothalamate (mL/min/1.73 m^2^) were determined.

Patients were stratified according to BMI (weight in Kg/height in m^2^) following the World Health Organization (WHO) obesity classification in grade I (BMI between 30 and 34.9 Kg/m^2^). grade II (BMI between 35 and 39.9 kg/m^2^) and grade III (BMI of 40 kg/m^2^ or more) [15].

GFR was measured by renal clearance of non-radiolabeled iothalamate, determined by high performance liquid chromatography (HPLC). The instrument used was a Gilson ^®^ HPLC with a Model 189 UV/Visible detector with a Phenomenex^®^ C18 column. In all cases, the procedure was carried out following a protocolized operations manual. Plasma samples were collected with heparin as an anticoagulant agent, and urine samples in sterile containers. The results are expressed adjusted to 1.73 m^2^ of body surface.

The determination of SCr was performed using the Jaffe kinetic method (Roche Diagnostics, Sussex, UK), traceable to the IDMS reference method, on a Modular P autoanalyzer. The calibration of the determination was performed with a commercial lyophilized calibrator for automated systems. All assays used participated in internal and external quality control programs (RIQAS, London, UK) and exceeded recommended assay quality specifications (acceptable total error). SCr values are expressed in mg/dL.

Estimated GFR was calculated from the following formulas: Cockcroft and Gault [16] adjusted for lean body mass (LBM_CG) [17], Salazar-Corcoran (SC) [18], Modification of Diet in Renal Disease adjusted for Isotope Dilution Mass Spectrometry (IDMS) standardization with 4 variables (MDRD4) and 6 variables (MDRD6) [19], Chronic Kidney Disease and Epidemiology version 2009 (CKD-EPI 2009) [20] and version 2021 (CKD-EPI 2021) [21], combined formula (CKD-MCQ) [22] between CKD- EPI 2009 and Mayo Clinic Quadratic equation (MCQ) [21], and finally the new AE (manuscript not yet published, presented and awarded at the XXII Argentine Congress of Nephrology [23]). This was developed using a quasi-likelihood model with an identity variance function (V(μ) = μ) and logarithmic link, with the intention of not having to transform the response variable Y, but rather predict it in its natural scale and without the need to assume a specific distribution for it [24]. For this model, six predictive variables were included: gender (male/female), age (years), single kidney (yes/no), square root of SCr (mg/dL), logarithm of urea (mg/dL), and serum albumin (g/L). The final equation was:

(1) $$\begin{array}{ll} \mathrm{AE}=\exp\;(6.3106 & -1.7656\times \sqrt{SCr}-0.0055\times age\\ & -0.0656\times \ln\;(urea\text{)}+0.060\times albumin+0.224\;if\;male\\ & -0.2052\;if\;single\;kidney \end{array}$$

The internal validation of this equation was carried out through 10-fold cross-validation [25], for this study only obese patients were used.

The performance of the GFR estimates of each formula (eGFR) was evaluated in relation to the GFR measured through the urinary clearance of iothalamate (mGFR, reference value). Bias was used (defined as the difference between eGFR and mGFR). A positive bias indicates an overestimation of mGFR and vice versa. As measures of position, the median (M), first quartile (Q1) and third quartile (Q3) of the bias were used. Accuracy was evaluated with the P30, defined as the percentage of observations whose eGFR differs from the mGFR by no more than 30% of the mGFR. For the agreement, the Pearson correlation coefficient (r) and the percentage of correct classification (%CC) were used, taking into account the percentage of patients well classified according to the eGFR in the 5 stages of chronic kidney disease [26], taking as reference the mGFR. Since the LBM_CG and SC equations predict creatinine clearance not adjusted for body surface area, mGFR not indexed to body surface area was used as the response variable to assess their performance, unlike the other equations. The scatter plot between mGFR and eGFR was made using the equation with the best performance.

To describe the characteristics of the patients, absolute (*n*) and relative (%) frequencies were used for the categorical variables, and for the continuous variables, medians (M) and Q1–Q3. To compare continuous variables, the Mann Whitney test was used and for categorical variables the Chi squared test or Fisher’s exact test, depending on the expected frequencies. All tests were two-tailed and a *p* value less than 0.05 was considered statistically significant.

The statistical analysis was carried out with the software: R Core Team (2021). A: A language and environment for statistical computing. R Foundation for Statistical Computing, Vienna, Austria and GraphPad Prism version 8.0.0 for Windows, GraphPad Software, San Diego, CA, USA.

The study was conducted in accordance with the Declaration of Helsinki, and approved by the Institutional Review Board of Hospital Privado Universitario de Córdoba (HP-4-264). Informed consent was obtained from all subjects involved in the study. The consent form included information on the procedure itself and on the possibility of later use of the data for research purposes.

## 3. Results

**Figure 1:**
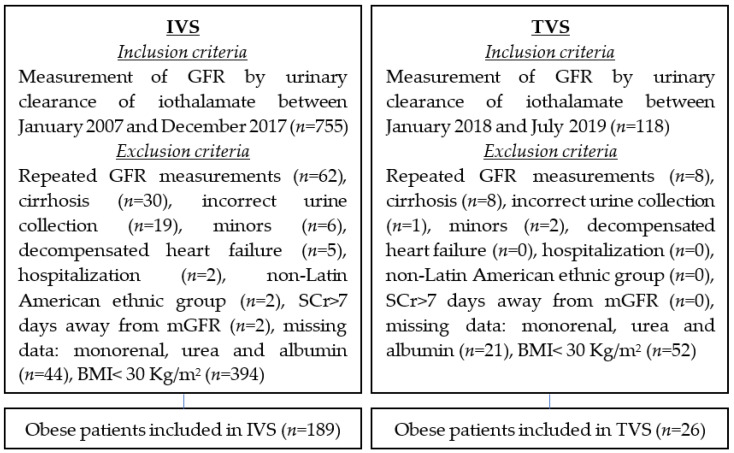
Flowchart with the inclusion and exclusion criteria in the internal validation sample (IVS) and in the temporary validation sample (TVS).

For the IVS, of the initial 755 subjects whose GFR was measured by urinary iothalamate clearance between 2007 and 2017, 566 were excluded for various reasons, and 189 subjects were included. For the TVS, of the 118 initial subjects whose GFR was measured by the same method between 2018 and 2019, 92 were excluded, and 26 were included (Figure 1).

The characteristics of the population are shown in Table 1. The median age was 50 (Q1–Q3 = 40.2–59.8) years, 52.1% were women and 47.9% men. The median BMI was 33.3 (31.7–37.5) kg/m^2^, but the maximum value was 72.3 kg/m^2^. The majority belonged to category I of obesity (60%), to a lesser extent to category II (25.1%) and category III (14.9%). 18.1% were diabetic, 47.1% hypertensive, and 9.3% single-kidney. Median SCr was 0.85 (0.71–1.1) mg/dL, urea was 31.1 (25.2–41.7) mg/dL, albumin was 4.2 (3.94–4.44) gr/L, and mGFR was 91.2 (70.2–116.2) mL/min/1.73 m^2^. There was a wide range in the mGFR (min-max = 5.6–173.1 mL/min/1.73 m^2^). 51.6% had an mGFR ≥90 mL/min/1.73 m^2^, 27.4% between 60 and 89.9 mL/min/1.73 m^2^, 14.4% between 30 and 59.9 mL/min/1.73 m^2^, 4.2% between 15 and 29.9 mL/min/1.73 m^2^ and 2.3% <15 mL/min/1.73 m^2^. There were no statistically significant differences between the characteristics of the subjects included in the IVS and in the TVS, with the exception of SCr levels, which were slightly lower in the IVS (0.84 vs. 0.97 mg/dL; *p* = 0.019).

Table 2 shows the performance of the equations to estimate the mGFR in the IVS. The equation with the best performance was AE, with the highest P30 (85.2%), correlation (0.86), %CC (74.4%) and a median bias closer to 0 (−0.4 mL/min/1.73 m^2^). The equations with the highest P30 after AE were CKD-EPI 2009 (84%), MDRD6 (83.5%) and CKD-EPI 2021 (82.4%). Those with the highest %CC after AE were CKD-EPI 2021 and CKD-MCQ (72.6%), and CKD-EPI 2009 (70.4%). CKD-EPI in its two versions and combined with MCQ obtained the same correlation as AE (0.86). CKD-EPI 2021 was the second that obtained a median bias closest to 0 (0.5 mL/min/1.73 m^2^). The equation that obtained the lowest performance was LBM_CG, followed by SC.

Table 3 shows the performance of the equations to estimate the mGFR in IVS, differentiating between the 3 degrees of obesity. In grade I obesity, AE was the equation with the best performance, with the highest P30 (87.4%), correlation (0.89), and a median bias closer to 0 (−0.4 mL/min/1.73 m^2^). Its %CC (74.8%) was second only to CKD-MCQ (75.7%). CKD-MCQ and CKD-EPI 2021 obtained the same correlation (0.89). CKD-EPI 2009 and MDRD6 were the ones that obtained the highest P30 after AE (86.4% and 85.4%). CKD-EPI 2021 obtained the second bias closest to 0 (0.6 mL/min/1.73 m^2^). The equation with the worst performance was LBM_CG. In grade II obesity, CKD-EPI 2021 obtained a slightly higher performance than AE, with higher P30 (84.4% vs. 82.2%), median bias closer to 0 (−1.9 vs. −3 mL/min/1.73 m^2^), equal correlation (0.88) and equal %CC (75.6%). In grade III obesity, AE was the equation with the highest P30 (82.1%) and %CC (67.9%). The equations with the highest correlation were CKD-EPI 2009 and 2021. The one that obtained a median bias closest to 0 was MDRD4 (−2.5 mL/min/1.73 m^2^). The performance of all the equations is reduced in grade III obesity. The only equation that obtained a P30 greater than 80% in the 3 degrees of obesity was AE. In general terms, the two equations with the worst performance were LBM_CG and SC.

**Figure 2:**
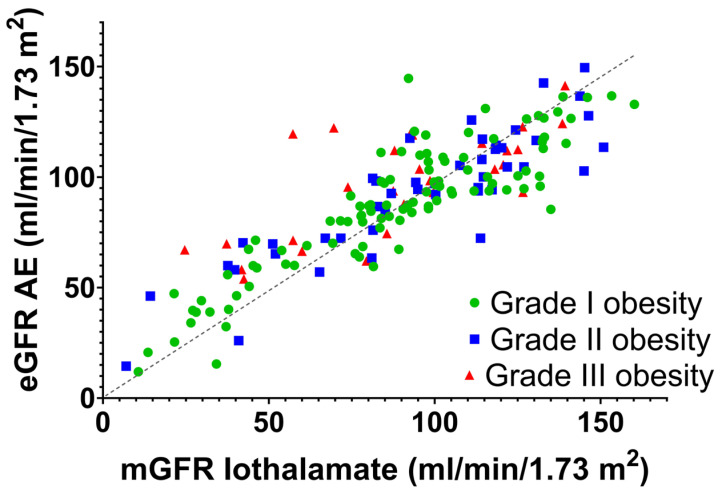
Scatter plot between the eGFR by Argentinian Equation (AE) and the mGFR by urinary clearance of iothalamate in the 3 degrees of obesity in IVS. The reference line is the identity line.

Figure 2 shows the scatter plot between the eGFR by AE and the mGFR by urinary clearance of iothalamate in the 3 degrees of obesity in IVS.

Table 4 shows the performance of the equations to estimate the mGFR in the TVS. In it, AE was also the one that obtained the highest P30 (88.5%, sharing with MDRD6), correlation (0.89, sharing with 3 other equations) and %CC (84.6%, sharing with CKD-EPI 2021). The median bias closest to 0 was obtained by CKD-EPI 2021 (1.1 mL/min/1.73 m^2^), although with greater dispersion than AE (Q1/Q3 = −6.8/12.1 vs. −4.3/8.6 mL/min/1.73 m^2^). In both the IVS and the TVS, a superior overall performance of AE can be observed over the other equations.

## 4. Discussion

This study evaluated the performance of currently available equations and the new AE for estimating GFR in obese patients. There is currently no consensus on the best equation to use in this population.

The SC equation was developed many years ago with obese patients with the intention of predicting creatinine clearance specifically in this population, since the equations available up to that time, such as CG, had poor performance in them [18]. Subsequently, it was observed that the use of the LBM instead of the current weight in the CG formula improves the prediction of creatinine clearance in obese subjects [27,28]. The studies in which these two equations were used to predict GFR using the mGFR with exogenous substances as the reference standard in obese patients, showed that the performance was inadequate, as in our study (either considering the prediction of the indexed mGFR or not indexed to body surface area) and many authors currently discourage their use [29,30]. The combination of CKD-EPI 2009 with MCQ had shown an improvement in the performance of each one separately, in subjects with obesity [22]. In our study, this combination improved the performance of MCQ alone, but not that of CKD-EPI 2009. When comparing the performance of CKD-EPI 2009 and MDRD4 in obese subjects in previous studies, the results are mixed [29,31,32,33,34,35,36,37]. In our study, considering the P30, there seems to be a small advantage of CKD-EPI in its two versions, over MDRD4 (except in grade III obesity). MDRD6 and CKD-EPI 2021 have not been evaluated in currently available studies. In our sample, MDRD with 6 variables improves the overall performance of MDRD with 4 variables (except in grade III obesity), and CKD-EPI 2021 has a median bias closer to 0 (except in grade III obesity) and higher % CC than CKD-EPI 2009.

The new AE obtained uniformly better performance than the other currently available equations (except in grade II obesity). This formula was developed using a different predictive model than the one used in the previous equations, which allows mGFR to be predicted directly on its natural scale, without the need to transform the response variable or assume a specific distribution beforehand [38]. We believe this is the most suitable for this type of estimation tools and the superior performance in the validation comparison demonstrated it. In this equation, unlike those currently in force, the presence of a single functioning kidney was incorporated as a predictor variable. These patients not only present less renal mass, but also suffer intraglomerular hemodynamic and structural adaptive changes [39,40], and changes in tubular creatinine secretion [41,42], which could modify the concentration of SCr and, therefore, the prediction of mGFR from it. This was the reason why we believe it is important to incorporate an adjustment coefficient in these subjects. As in MDRD6, AE incorporated urea and albumin as predictive variables. In subjects with changes in muscle mass, in which Scr is not a good predictor [13], these variables that do not directly depend on muscle mass could generate an extra fit to the model.

AE had 2 validations, in the internal validation the 10 fold cross-validation method was used, unlike the current equations, which is a modern statistical method of resampling, recommended and considered the best method for internal validation at present [43]. Secondly, a temporary validation was carried out, with totally independent subjects and from a later period. This validation is considered as an intermediary between internal and external validation, although some authors consider it as a subtype of external validation [43]. The comparisons of the performance of the previous equations and AE were always made separately in each of the validation samples, that is, without mixing the IVS subjects with those of the TVS. This double validation allows reaffirming the performance of the equation, although in the TVS it was not possible to perform a sub-analysis with the degrees of obesity due to the small number of subjects.

Some authors suggest that the P30 is the best metric to compare the performance of different equations, since it combines bias with precision [44,45]. A P30 value of 80 to 90% is considered acceptable for the evaluation of the GFR in many circumstances of clinical practice [21]. When it is required to know the GFR with greater precision, the estimates do not replace the measurement. AE obtained a P30 greater than 80% in both validation samples, and even in the 3 degrees of obesity. Although, as in previous studies, it was possible to observe that in grade III obesity all the equations reduce their performance [22,31], the only equation that obtained a P30 > 80% in patients with this degree of obesity was AE.

Being a cross-sectional study, we did not evaluate the changes over time in the GFR and the variables associated with it, such as weight fluctuations and glycemic parameters [46,47]. We only set out to assess the ability to estimate the measured GFR of the different equations at one point in time. We also did not evaluate the performance of the equations that use cystatin C, since none of our patients had this measurement. In our environment there is little availability of cystatin C and it is expensive, so it is unlikely that the clinical use of equations that require this marker will be used in the short and medium term [48].

The weaknesses of this study are: it was carried out in a single center, with a sample that does not represent all obese subjects in the population, and without the use of classical external validation.

The strengths of the study are: the inclusion of patients with a wide range of GFR, the use of mGFR as a reference standard, the availability of SCr standardized to the IDMS method, and the double validation of a new equation developed in Latin America with novel statistical strategies.

## 5. Conclusions

The new AE obtained a better overall performance than the current equations to estimate the mGFR in subjects with obesity in our environment. It could be useful in this type of patients in some circumstances, although more studies are needed to confirm it. Conclusions from this study may not be generalizable to all populations of obese patients since they were derived from a study in a single center with a very specific ethnic mixed population.

## Figures and Tables

**Table 1:** Characteristics of all the patients, in the internal validation sample (IVS) and in the temporary validation sample (TVS).

Characteristics	All (*n* = 215)	IVS (*n* = 189)	TVS (*n* = 26)	*p*
Age (years) ^a^	50 (40.2–59.8)	50 (40.2–59.8)	50.5 (41–59)	0.840
Female gender ^b^ Male gender ^b^	112 (52.1) 103 (47.9)	100 (52.9) 89 (47.1)	12 (46.2) 14 (53.8)	0.518
Body mass index (kg/m^2^) ^a^	33.3 (31.7–37.5)	33.3 (31.6–37.7)	32.7 (31.7–36.5)	0.605
Grade I obesity ^b^ Grade II obesity ^b^ Grade III obesity ^b^	129 (60) 54 (25.1) 32 (14.9)	112 (59.2) 47 (24.9) 30 (15.9)	17 (65.4) 7 (26.9) 2 (7.7)	0.604
Diabetes ^b^	37 (18.1)	34 (19.1)	3 (11.5)	0.427
Hypertension ^b^	96 (47.1)	86 (48.3)	10 (38.5)	0.347
Single kidney ^b^	19 (9.3)	17 (9.6)	2 (7.7)	1
Creatinine (mg/dL) ^a^	0.85 (0.71–1.1)	0.84 (0.69–1.09)	0.97 (0.82–1.18)	0.019
Urea (mg/dL) ^a^	31.1 (25.2–41.7)	31.2 (25.2–41.7)	30.3 (26.1–41.1)	0.751
Albumin (g/L) ^a^	4.2 (3.94–4.44)	4.19 (3.94–4.43)	4.34 (3.93–4.51)	0.303
mGFR (mL/min/1.73 m^2^) ^a^	91.2 (70.2–116.2)	92.6 (71.6–117.3)	80.2 (70.2–96.2)	0.109
mGFR < 60 mL/min/1.73 m^2 b^	45 (20.9)	39 (20.6)	6 (23.1)	0.774

^a^ Values are expressed as median (Q1–Q3). ^b^ Values are expressed as *n* (%). Abbreviations: mGFR, measured glomerular filtration rate.

**Table 2:** Performance of the different equations to estimate the mGFR in the IVS (*n* = 189).

Equations	Bias (Q1/Q3)	P30 (%)	r (95% CI)	%CC
LBM_CG	−22.6 (−36.3/−4.5)	65.3	0.74 (0.67–0.80)	55.7
SC	5.1 (−7.8/21.9)	76.1	0.73 (0.65–0.79)	64.8
MDRD4	−8.4 (−18.9/6.3)	81.8	0.81 (0.76–0.86)	63.1
MDRD6	−7.3 (−18.4/5.8)	83.5	0.83 (0.78–0.87)	63.1
CKD-MCQ	3.7 (−8.6/12.2)	78.9	0.86 (0.82–0.89)	72.6
CKD-EPI 2009	−4.4 (−15.8/7.7)	84	0.86 (0.81–0.89)	70.4
CKD-EPI 2021	0.5 (−11.3/11)	82.4	0.86 (0.82–0.89)	72.6
AE	−0.4 (−11.5/10.2)	85.2	0.86 (0.82–0.89)	74.4

Abbreviations: Q1, first quartile; Q3, third quartile; r, Pearson correlation coefficient; 95% CI, 95% confidence interval; %CC, percentage of correct classification; LBM_CG, Cockcroft and Gault adjusted for lean body mass; SC, Salazar-Corcoran; MDRD4-6, Modification of Diet in Renal Disease with 4 and 6 variables; CKD-MCQ, combined formula between CKD- EPI 2009 and Mayo Clinic Quadratic equation; CKD-EPI 2009–2021, Chronic Kidney Disease and Epidemiology version 2009 and 2021; AE, Argentinian Equation.

**Table 3:** Performance of the different equations to estimate the mGFR in the IVS differentiating between the 3 degrees of obesity (*n* = 189).

Equations	Bias (Q1/Q3)	P30 (%)	r (95% CI)	%CC
Grade I obesity (*n* = 112)				
LBM_CG	−21.9 (−37.5/−5)	63.1	0.83 (0.75–0.88)	54.4
SC	1.8 (−10.6/15.4)	79.6	0.83 (0.76–0.88)	66
MDRD4	−8.7 (−20.9/5)	83.5	0.85 (0.79–0.90)	63.1
MDRD6	−6.7 (−18.4/5.2)	85.4	0.87 (0.81–0.91)	62.1
CKD-MCQ	2.9 (−8.6/10.2)	81.6	0.89 (0.84–0.92)	75.7
CKD-EPI 2009	−4.6 (−16.3/6.8)	86.4	0.88 (0.83–0.92)	71.8
CKD-EPI 2021	0.6 (−11.7/9.9)	83.5	0.89 (0.84–0.92)	72.8
AE	−0.4 (−11.2/8.7)	87.4	0.89 (0.84–0.92)	74.8
Grade II obesity (*n* = 47)				
LBM_CG	−26.1 (−39/−9.1)	62.2	0.77 (0.61–0.87)	60
SC	4.5 (−11.6/21.8)	77.8	0.77 (0.62–0.87)	66.7
MDRD4	−13.2 (−18.9/5.2)	77.8	0.81 (0.68–0.89)	64.4
MDRD6	−8.9 (−19.5/3.5)	82.2	0.86 (0.77–0.92)	68.9
CKD-MCQ	2 (−9/9.4)	82.2	0.89 (0.80–0.94)	73.3
CKD-EPI 2009	−6.1 (−17.3/4.2)	84.4	0.88 (0.80–0.93)	75.6
CKD-EPI 2021	−1.9 (−12.8/7.1)	84.4	0.88 (0.80–0.93)	75.6
AE	−3 (−14.6/7.4)	82.2	0.88 (0.79–0.93)	75.6
Grade III obesity (*n* = 30)				
LBM_CG	−16.2 (−29/5.7)	78.6	0.64 (0.34–0.82)	57.1
SC	23.4 (8.7/44.7)	60.7	0.61 (0.31–0.80)	57.1
MDRD4	−2.5 (−13.1/13.3)	78.6	0.69 (0.43–0.85)	64.3
MDRD6	−4.4 (−13.8/12.1)	78.6	0.64 (0.35–0.82)	60.7
CKD-MCQ	9.9 (−2.7/30.4)	64.3	0.74 (0.51–0.87)	64.3
CKD-EPI 2009	2.6 (−9.3/18.7)	75	0.76 (0.54–0.88)	60.7
CKD-EPI 2021	6.3 (−5.4/22.5)	75	0.76 (0.55–0.89)	67.9
AE	4.2 (−10.1/22.3)	82.1	0.71 (0.46–0.86)	67.9

Abbreviations: Q1, first quartile; Q3, third quartile; r, Pearson correlation coefficient; 95%CI, 95% confidence interval; %CC, percentage of correct classification; LBM_CG, Cockcroft and Gault adjusted for lean body mass; SC, Salazar-Corcoran; MDRD4–6, Modification of Diet in Renal Disease with 4 and 6 variables; CKD-MCQ, combined formula between CKD- EPI 2009 and Mayo Clinic Quadratic equation; CKD-EPI 2009–2021, Chronic Kidney Disease and Epidemiology version 2009 and 2021; AE, Argentinian Equation.

**Table 4:** Performance of the different equations to estimate the mGFR in the TVS (*n* = 26).

Equations	Bias (Q1/Q3)	P30 (%)	r (95% CI)	%CC
LBM_CG	−21.5 (−30.9/−10.6)	61.5	0.89 (0.78–0.95)	69.2
SC	1.6 (−10.5/13.6)	84.6	0.89 (0.77–0.95)	73.1
MDRD4	−11.3 (−16.4/0.6)	80.8	0.87 (0.73–0.94)	65.4
MDRD6	−6.6 (−14.1/2.2)	88.5	0.86 (0.71–0.94)	65.4
CKD-MCQ	5.4 (−1.1/12.2)	84.6	0.89 (0.77–0.95)	80.8
CKD-EPI 2009	−3.9 (−10.9/7.1)	84.6	0.87 (0.74–0.94)	73.1
CKD-EPI 2021	1.1 (−6.8/12.1)	84.6	0.89 (0.76–0.95)	84.6
AE	2.6 (−4.3/8.6)	88.5	0.89 (0.78–0.95)	84.6

Abbreviations: Q1, first quartile; Q3, third quartile; r, Pearson correlation coefficient; 95% CI, 95% confidence interval; %CC, percentage of correct classification; LBM_CG, Cockcroft and Gault adjusted for lean body mass; SC, Salazar-Corcoran; MDRD4-6, Modification of Diet in Renal Disease with 4 and 6 variables; CKD-MCQ, combined formula between CKD- EPI 2009 and Mayo Clinic Quadratic equation; CKD-EPI 2009–2021, Chronic Kidney Disease and Epidemiology version 2009 and 2021; AE, Argentinian Equation.

## Data Availability

Not applicable.
